# Prevalence of Prediabetes, Diabetes, and Its Associated Risk Factors among Males in Saudi Arabia: A Population-Based Survey

**DOI:** 10.1155/2018/2194604

**Published:** 2018-04-24

**Authors:** Khaled K. Aldossari, Abdulrahman Aldiab, Jamaan M. Al-Zahrani, Sameer H. Al-Ghamdi, Mohammed Abdelrazik, Mohammed Ali Batais, Sundas Javad, Shanila Nooruddin, Hira Abdul Razzak, Ashraf El-Metwally

**Affiliations:** ^1^Family & Community Medicine Department, College of Medicine, Prince Sattam Bin Abdulaziz University, Al-Kharj, Saudi Arabia; ^2^Department of Medicine, Oncology Division, College of Medicine, King Saud University, Riyadh, Saudi Arabia; ^3^General Surgery Department, College of Medicine, Prince Sattam Bin Abdulaziz University, Al-Kharj, Saudi Arabia; ^4^College of Medicine, King Saud University, Riyadh 29391, Saudi Arabia; ^5^Epidemiology & Biostatistics Department, College of Public Health & Health Informatics, King Saud Bin Abdulaziz University for Health Sciences, Riyadh, Saudi Arabia; ^6^Aga Khan University Hospital, Karachi, Pakistan; ^7^Ministry of Health and Prevention, Dubai, UAE; ^8^Department of Epidemiology, School of Health Sciences, University of Tampere, Tampere, Finland; ^9^College of Public Health & Health Informatics, King Saud Bin Abdulaziz University for Health Sciences, Riyadh, Saudi Arabia; ^10^King Abdullah International Medical Research Center (KAIMRC), Riyadh, Saudi Arabia

## Abstract

**Objectives:**

The study aims at determining the prevalence of prediabetes and diabetes and at ascertaining some concomitant risk factors among males in Saudi Arabia.

**Methods:**

A population-based cross-sectional study including 381 Saudi adult males from different institutions was recruited. Odds ratios for diabetes risk and risk factors were calculated using log-binomial and multinomial logistic regression, using STATA version 12.

**Results:**

The participants included 381 diabetic males with a median age of 45 years, average body mass index of 25 ± 40 kg/m^2^, whereas waist circumferences ranged from 66 to 180 cm in the male study population. In addition, 27.82% had normal BMI, 32.28% were overweight, and 36.22% were obese. Around 36% had higher waist circumference, that is, >102 cm. Age, BMI, marital status, and educational attainment were statistically significant predictors for prediabetes and diabetes.

**Conclusion:**

This study found that the prevalence of diabetes and prediabetes was 9.2% and 27.6%, respectively, for male Al-Kharj study population. The factors that increase the risk of diabetes and prediabetes include older age, obesity and overweight, being married, smoker, and having a civilian job and less education. All these factors were found statistically significant except smoking status and job type. In order to evaluate the causal relationship of these factors, prospective studies are required in future.

## 1. Introduction

Diabetes mellitus (DM) is one of the five principal causes of death universally [1]. In Saudi Arabia, primary epidemiological diabetes features are not different. The diabetes mellitus prevalence among adult population has reached 23.7%, a percentage being the highest across the globe [2, 3]. The diabetes burden upon the population of Saudi's remains to be on the rise, the more individuals diagnosed newly with this condition, the more a population is at risk to develop amputations, nervous system disease, kidney disease, blindness, hypertension, stroke, heart disease, dental disease, and amputations [4]. Awareness and knowledge regarding the diabetic condition, its management, complications, and risk factors are crucial steps for better quality of life and its control [5]. A recognized basis to prevent this condition is to classify common modifiable risk factors that have an ultimate impact on the morbidity on top of developing community-based programs for its control and prevention. Normally, by the time an individual is diagnosed, they have a tendency to develop various complications, such as ischemic heart disease (a macroangiopathic procedure) or retinopathy (a microangiopathic process) [6]. In this manner, the fact that some of the risks (such as environmental, medical, and lifestyle factors) might precipitate diabetes specifically in the individuals predisposed genetically. Due to this, diabetes leads to consequences, such as hypertension. This is a fundamental disease trigger that warrants early intervention to control the concern of diabetes within a community [7].

The screening and its effectiveness is primarily based on the settings wherein it is undertaken. Programs based on community screening specifically on a larger scale may be targeted poorly; explicitly, it might fail to reach the groups mostly at risk as well as unsuitably testing those who are at a reduced risk or precisely those diagnosed already. World Health Organization (WHO) predominantly recommends the screening for diabetes in few high-risk groups, such as older age categories [8]. Similarly, “United States Preventive Services Task Force” has recommended screening of diabetes in adults devoid of precise symptoms and in individuals with BP higher than 135/80 mmHg [9].

Commonly, a diversity of techniques such as questionnaires based on risk assessment as well as analysis founded on the concentration of plasma glucose measurement was undertaken on the venous samples with enzymatic assay procedures, for screening of prediabetes that are in practice [10]. Pencil and paper tests, for example, the American Diabetes Association's risk test is helpful for the purpose of education but does not accomplish well as a stand-alone tests. Prediabetes screening analysis comprises of, but is not limited to, casual plasma glucose level, 75 g oral glucose tolerance test (OGTT), and fasting plasma glucose (FPG) [11]. It can also be termed as intermediate or borderline hyperglycemia or and can be diagnosed with an HbA1c of 5.7% to 6.4% [12]. The American Diabetes Association previously equated prediabetes with the WHO's intermediate hyperglycemia, but recently added borderline levels of HbA1c as another indicator [13]. The Expert Committee on Diagnosis and Classification of Diabetes Mellitus identified an intermediate group of people with glucose levels that do not meet criteria for diabetes, yet having higher than normal. These individuals were defined as having impaired fasting glucose (IFG) [FPG levels 100 mg/dl (5.6 mmol/l) to 125 mg/dl (6.9 mmol/l)] or impaired glucose tolerance (IGT) [2 h values in the OGTT of 140 mg/dl (7.8 mmol/l) to 199 mg/dl (11.0 mmol/l)]. Individuals with IFG and/or IGT were referred as having prediabetes, showing the relatively high risk for the future development of diabetes [12]. Globally, its prevalence has been found to be increasing, and by 2030, it can rise up to >470 million people suffering from it. [14] Additionally, several cutoff points and thresholds at different community and healthcare settings, together with the portable capillary blood assessments, are also used. Most essentially, the presence of positive prediabetic state demonstrates a higher risk for developing the condition of DM [11, 15].

Similar statistics regarding the increasing trend of diabetes and prediabetes in the world have also been observed in Saudi Arabia. As per the WHO country profile 2016, 14.4% of Saudi population has diabetes, while prevalence in males is 14.7% [16]. In 2015, the prevalence of prediabetics was found to be 9.0% in Jeddah with 9.4% in men, while for diabetes, it was 12.1% with 12.9% adult male population suffering from it [17]. Another study conducted in Saudi population revealed that the diabetes prevalence in their study was found to be 25.4%, while impaired fasting glucose (IFG) was 25.5%. The strongest risk factors were age > 45 years, high triglycerides levels, and hypertension [18].

This study therefore aims to determine an alarming diabetes and prediabetes prevalence among Saudi adult population, along with its associated risk factors. Since the data regarding the epidemiological aspects of diabetes is scarce in the Saudi population [19], there is a need to discover various common risk factors and their association with diabetes and prediabetes in order to eliminate the complications and adverse health effects of this disease.

## 2. Methodology

### 2.1. Study Design

The study is a population-based cross-sectional study.

### 2.2. Settings

Al-Kharj is a city located around 77 km south of Riyadh within Al-Kharj Governorate in Central Saudi Arabia with a population density of 376,000, being one of the major hubs with up-to-date administration and economic importance possessing significant natural resources, important geographical location, population diversity, as well as a population with various racial and ethnic backgrounds. The results concluded from the population residing in this region can have good generalizability on the reference population of the adult males within the country.

### 2.3. Inclusion and Exclusion Criteria

The selection criteria included the participants aged 18 years of age or above. The eligibility criteria for the study population included Saudi adult males willing to participate as well as sign a consent form. The exclusion criteria included non-Saudi residents, who were younger than 18 years of age or those who were not willing to participate and did not sign the consent form. However, data were collected from females but were excluded from the analysis.

### 2.4. Data Collection and Sample Size

Data was collected from January 2016 to June 2016 with a total sample size of 1200, with a response rate of 85%. Complete data was available for 1019 individuals where 381 were males and 638 were females. Due to difference in age structure, separate analysis was performed for males and females. From previous studies carried out in Saudi Arabia at national level, we know the prevalence of diabetes was found to be 25% (which is 0.25 proportion). We would like to estimate the prevalence of diabetes within 5% of the true prevalence in our study population. This is the minimum sample required in order to estimate true prevalence in Al-Kharj population.

(1) $$\begin{array}{c} N=0.25\;\left(1-0.25\right)\;{\left(\frac{1.96}{0.05}\right)}^{2}=288.12. \end{array}$$

A multi-stage sampling method was used. Samples of different governmental and private institutes were selected through a cluster sampling technique. The total population of these institutions was divided into groups called clusters after obtaining a list of participants in each selected institute. Then samples of participants were selected using simple random sampling from each of the group (cluster).

### 2.5. Materials/Instruments

For data collection, an interview was conducted from the respondents using a 20-item Arabic questionnaire by two trained physicians. The questionnaire was designed after a deep evaluation of the literature published. The material gathered included first part: demographics (like age, education, employment, and marital status), second part (smoking, chronic medical illness, chronic pain, and GHQ-12), and the last part consists of physical parameters including weight, height, and waist circumference. All participants completed a self-administrated questionnaire, followed by a physical examination and blood test.

### 2.6. Physical Measurement

Nurses who were well trained gathered the anthropometric weight measurements, waist circumference, and height. Height was measured to the nearest 0.5 cm without shoes, waist circumference measured to the nearest 0.5 cm using measuring tape, while weight was measured to the nearest 100 g with the subject being either without shoes or lightweight clothes. A Health O Meter Digital Scale (made in USA), which reads to the nearest 100 g, was utilized as the weighing scale. A specific scale was utilized to weigh all the participants. This scale was calibrated regularly, as well as zero was assured prior to taking the weight of any participant.

### 2.7. Body Mass Index

Body mass index (BMI) was calculated as weight in kilograms divided by height in meters squared (kg/m^2^) for all participant being studied. Normal (BMI < 25), overweight (25 < BMI < 30), and obese (BMI > 30) groups were composed based on widely used cutoff values for assessing obesity [20].

### 2.8. Waist Circumference

Waist circumference (WC) is used as surrogate for abdominal obesity and WC value less than 102 cm is considered normal WC in men while less than 88 cm in women [21].

### 2.9. Blood Test

Blood samples were collected from each participant by trained nurses or phlebotomist for phlebotomy. The patient is given a unique ID (barcode). The specimen label and the forms are checked if they are the same. Two tubes were used: one tube for hemoglobin A1c while the other tube for chemistry, then gentle rolling is applied at roller mixer to prevent clotting. Any clotted samples or critical results were reported and participants were contacted immediately for another sample. The tubes were collected in a special container containing ice for more care. Once finished by the phlebotomist, the samples were sent to the central laboratory within 1-2 hours of duration. The machine is checked for the calibration and control if it is in good condition, and during that time, samples are run. For the test procedure, the data of all the samples/specimen should be encoded in the machine named *Beckman Coulter* to work it automatically. The results are checked and if any abnormal results, we will repeat it manually. The heparin plasma samples are used for the chemistry analysis. The heparin vacutainers are separated from the remaining. Then the vacutainers are arranged according to the barcode numbers and kept in the centrifuge. Samples are centrifuged for 5 minutes at 4000 rpm for the separation of plasma. Once the plasma is separated, it is used for the test procedure. For the test procedure, calibration (check and control) of the test is required. Samples are programmed into the machine named *Dimension Xpand Plus* accordingly and after the test is finished, results are collected.

### 2.10. Prediabetes and DM Definition

Prediabetes was defined using HbA1c cutoff level of 5.7–<6.5%, while diabetes mellitus (DM) is ≥6.5%, according to the American Diabetes Association 2016 [22]. Participants with previously diagnosed diabetes were also included during analysis to estimate true prevalence in the population. HbA1c was chosen as a method to define diabetes and prediabetes because HbA1c does not require fasting and blood can be drawn at any time of the day. Subsequently, this method was chosen due to convenience of participants and to be able to have bigger sample size. This is a standard method used in government hospitals in Saudi Arabia to diagnose diabetes.

### 2.11. Data Analysis

The general and baseline characteristics between diabetic and nondiabetic individuals were reported using mean (standard deviation) for continuous variables and percentage (sample size) for categorical variables. Significance of differences was also assessed using *χ*^2^ test for categorical variables and using *t*-test or ANOVA for continuous variables. Additionally, prevalence (%) of various risk factors in participants with diabetes and prediabetes was reported. Standardization for prediabetes and diabetes prevalence was computed using general authority of statistics survey 2016 data for adjusting to national population and for comparative purposes. We calculated odds ratios (OR) using log-binomial regression and multinomial logit regression for estimating diabetes risk and its association with different risk factors such as anthropometric measures and lifestyle factors. All *p* values were considered to be two tailed, with a *p* value of less than 0.05 representing statistical significance and statistical software StataCorp STATA version 12 was used for analysis.

### 2.12. Ethical Approval

The study protocol was approved by the local Institutional Review Board. Confidentiality of the gathered information of the participants' and clinical data had been assured, and a written informed consent from each respondent was gathered as a personal permission to take part in the study.

## 3. Results

Baseline general characteristics were described using mean (SD) calculated for continuous variables and percentage for categorical variables by their diabetes status and for total population in Table 1. Additionally, *p* value was calculated using *t*-test or ANOVA for continuous variable and *χ*^2^ test for categorical data to assess significance of difference. The study population age ranged from 18 to 60 with average age 31.42 (SD = 9.4). There were 25.8% males aged 18–24 years, 63% aged 25–44 years, and 11.3% aged 45–60 years. Individuals with diabetes had average age of 43.83 (SD = 11.35), while prediabetes individuals had average age of 33.44 (SD = 8.31). 70.5% of prediabetes cases were found in 25–44 age, while 40% and 51.4% of diabetes cases were found in 25–44 and 45–60 aged individuals, respectively. In our findings, prediabetes is more prevalent in middle age (25–44 age group) and diabetes in 45–60 age group as shown in Table 2. The prevalence of diabetes is shown to be increasing proportionally with increasing age (*p* < 0.0001), whereas prediabetes reaches a limiting point at 25–44 age group. We found prevalence ratios of 8.3 and 1.1 for having prediabetes compared to diabetes given an individual fall in age group 45–60 (versus 18–24) and age group 25–44 (versus 18–24), respectively.

Males had average of 5.69 glycosylated hemoglobin (HbA1c) levels (SD = 1.12, range = 4.4–13.3), whereas 355 had HbA1c levels less than 6.5% and 10 males had HbA1c levels more than 10%. We found a total of 35 diabetic cases and 105 prediabetic cases in Al-Kharj population of Saudi Arabia. We found 14 individuals with diabetes status who were unaware of their diabetes status and 21 self-reported diabetes cases. Regardless of awareness of diabetes status, 12 out of 21 had poor management of their HBA1c level, while only 9 had their HBA1c under recommended levels.

Body mass index ranged from 15 to 57 kg/m^2^ with average BMI 28.8 (SD = 6.94), whereas waist circumferences ranged from 66 to 180 cm in the male study population and 25 men had body mass index higher than 40 kg/m^2^. Overall study participants with normal body mass index were 31.5%, 32.3% overweight, and 36.2% obese. We found that 137 males (36%) had high waist circumference (WC > 102 cm) and none of the males had high waist circumference but normal BMI. To establish relationship between measurements of abdominal and general obesity, correlation between BMI and WC was found to be statistically significant at 0.05 with *r* = 0.88. Diabetic individuals had average BMI value of 30.5 (SD = 5.7), indicating prevalence of obesity in diabetic individuals with 54.3% obese and 28.6% overweight. Similarly, prediabetic individuals had average BMI value of 31.02 (SD = 6.74), indicating high prevalence of obese and overweight individuals with 49.5% obese and 31.4% overweight. The prevalence of obesity for diabetes and prediabetes is shown to be increasing proportionally with increasing BMI (*p* < 0.0001) as shown in Table 2. Overall study populations with normal WC were 64% and 36% with high WC, whereas 52.4% prediabetic and 40% diabetic individuals had high abnormal WC. Diabetic and prediabetic male participants had higher WC and BMI on average and were older in age as compared to nondiabetics and difference was found to be statistically significant at *p* value less than 0.005.

Crude prevalence for diabetes and prediabetes was 9.2% and 27.6%, respectively, as shown in Table 2 for male Al-Kharj study population. Additionally, age-specific prevalence and other trait-specific prevalence with 95% confidence interval are tabulated in Table 2. The prevalence of diabetes is shown to be increasing proportionally with increasing age as 3.1%, 5.8%, and 41.9% for age group 18–24, 25–44, and 45–60, respectively. Similarly, prevalence of prediabetes is demonstrated to increase proportionally with increasing age but reaches limiting point at age group 25–44 as prevalence of prediabetes 18.4%, 30.8%, and 30.2% for age group 18–24, 25–44 and 45–60, respectively. Prevalence of diabetes is shown to be increasing proportionally with increasing levels of BMI as 5%, 8.1%, and 13.8% for normal, overweight, and obesity levels, respectively. Prevalence of prediabetes is shown to be also increasing proportionally with increasing levels of BMI as 16.7%, 26.8%, and 37.7% for normal, overweight, and obesity levels, respectively. High WC had 15.3% prevalence of diabetes and 36.5% prevalence of prediabetes. Diabetes found to be more prevalent in older age, higher BMI, and high waist circumference groups, whereas prediabetes found to be more prevalent in 25–60 age group, higher BMI (overweight and obese), and high waist circumference (>102 cm) groups. We found prevalence ratios of 1.7, 1.2, and 1.01 for having prediabetes compared to diabetes given an individual with high WC (versus WC < 102 cm), obese (versus normal BMI), and overweight (versus normal BMI), respectively.

Standardization for age and sex based on national Saudi Arabia survey conducted by general authority of statistics (https://www.stats.gov.sa/en/43) in order to adjust prevalence of prediabetes and diabetes to national population of Saudi Arabia for comparison purposes is shown in Table 3. Overall 29.46% and 14.36% prevalence for prediabetes and diabetes, respectively, adjusted to national Saudi population of 18–60 years age group. For overall 25–60 years age group, 19.04% and 34.46% prevalence for prediabetes and diabetes, respectively, adjusted to national Saudi population. Clearly, it shows transition of prediabetes cases to diabetes cases when comparing diabetes and prediabetes prevalence for 18–60 and 25–60 age years. Diabetes prevalence for males 18–60 years age was found to be 13.97% with 14.76% for females and prediabetes prevalence for males was found to be 27.17% in contrast to 31.84% for females.

Overall study participants with current smoking status were 25.2%, whereas 7.9% were reported to be ex-smoker. We found that 6.25% and 35.4% of current smoker had diabetes and prediabetes, respectively, whereas 13.3% and 23.3% of ex-smoker had diabetes and prediabetes, respectively. No clear indication for smoking association was found with diabetes status as trend was found to be nonsignificant (*p* value = 0.289) for smoking status and having diabetes.

Out of 381 males, 57.5% (*N* = 219) were married, whereas 42.5% (*N* = 162) were single. But 12.3% and 35.2% of individuals with married marital status had diabetes and prediabetes, respectively, whereas 4.9% and 17.3% with single marital status had diabetes and prediabetes, respectively, as shown in Table 2. Clearly, individuals being married more likely to have diabetes and prediabetes when compared to single (*p* value < 0.0001). We also found prevalence ratio of 1.2 for having prediabetes in comparison to diabetes given an individual being married compared to being single.

The level of education of the male study population was 65% university level or higher educational level, whereas 29% had secondary level education or less. Out of 381 male participants, 37% of the diabetic cases were university or postgraduate educated, whereas 57% of the prediabetic cases had university level or postgraduate level education. 54.6% and 36.4% of individuals with primary education had diabetes and prediabetes, respectively, whereas 5.2% and 24.1% with university or postgraduate education had diabetes and prediabetes, respectively, as shown in Table 2. The prevalence of diabetes and prediabetes is shown to be decreasing proportionally with education attainment level (*p* < 0.0001).

In this study population, unemployment rate was 8.4%. Only 91.1% of the males were employed in the civilian sector, while 0.52% were employed as soldiers. 94.3% of individuals who had diabetes were holding civilian job, whereas only 5.7% diabetics were unemployed and none of the soldiers had diabetes. Similarly, 92.4% of prediabetic individuals were holding civilian job, whereas 5.7% and 1.9% prediabetics were unemployed and soldiers, respectively. Prevalence for diabetes and prediabetes for civilian job was found to be 9.5% and 27.9% as shown in Table 2. Likewise, 6.3% and 18.8% prevalence was found for diabetes and prediabetes among unemployed individuals. Diabetic and prediabetic individuals holding civilian job are more likely to have diabetes when compared to unemployed (*p* value < 0.0001). We also found prevalence ratio of 1.02 having diabetes compared to prediabetes given an individual holding civilian job compared to being unemployed.

Table 4 demonstrates the unadjusted and age-adjusted relationships between the number of traits and diabetes status using log-binomial regression for diabetic and nondiabetic and using multinomial logit regression modeling for prediabetic and diabetic levels to measure odds ratio (OR). Age, obesity, waist circumference, marital status, and secondary and university or higher education level were significant before adjustment for diabetes as compared to overall nondiabetes in log-binomial regression model. Following age adjustment, none of the risk factors were significant at *p* value < 0.05 for diabetes compared to total nondiabetes.

For prediabetes compared to nondiabetes, age, overweight, obesity, waist circumference, marital status, and university or higher education level were significant before adjustment. However following adjustment, only obesity, waist circumference, and marital status remained significant for prediabetes compared to nondiabetics. Similarly, for diabetes compared to nondiabetes in multinomial logit regression model, 45–60 age group, overweight, obesity, waist circumference, marital status, and secondary and university or higher education level were significant before adjustment. Following age adjustment, only waist circumference (high versus low), university or higher education level (versus primary education), and civilian job type (versus unemployed) were found to be significant at *p* value < 0.05 for diabetes compared to nondiabetes. Our result findings demonstrate that age, obesity (versus normal BMI), overweight (versus normal BMI), being married (versus single), and current or ex-smokers (versus nonsmokers) increase the risk of having prediabetes and diabetes, whereas being university or highly educated compared to primary education level and holding civilian job (versus unemployed) decreases the risk of diabetes and prediabetes.

## 4. Discussion

One of the commonest noncommunicable chronic diseases is diabetes mellitus with high worldwide prevalence in the current situation. Along with this, the prediabetic stage has also become prevalent. It carries an enormous environmental and genetic background. It is estimated that in 2025, 300 million people will be affected and so it continues to be worldwide-growing epidemic [22]. Our study contributed in evaluating the risk factors present among adult male population in Saudi Arabia as well as various other determining and contributing factors for prediabetes as well. In our study, education attainment, total obesity, marital status, and age above 45 were found to be significant predictors of prediabetes and diabetes as compared to nondiabetic with the exception of job type and smoking status.

However, various other risk factors for diabetes were also identified in men including high systolic blood pressure, smoking, alcohol intake, and men who lived alone, as described by a study [23]. As rationalized by our study, men were more prone to be diabetic because of having risk factors enumerated in our study. Several other studies were of the same idea that a higher prevalence of type 2 diabetes was observed in men than in women [24–27]. Additionally, less physical activity, presence of CVD, hypertension, central adiposity, deprived nutrition, and a first degree relative with type 2 diabetes [28] and hypertriglyceridemia [29] were also discussed as significant risk factors for diabetes and prediabetes.

Regarding the risk factors for diabetes, obesity (measured with BMI and WC) was found to have significant association with the occurrence of diabetes in our study. Participants with normal body mass index were 27.82%, while 32.28% were overweight and 36.22% were obese. We also found that 36% males had high waist circumference (WC > 102 cm). Contribution of obesity towards diabetes has been supported by previous published literature. Recent findings also suggest that the incidence of obesity is increasing more in men as compared to women. [30–33]. A study described that during the process of analysis, additional inclusion of BMI did not alter the association between men and type 2 diabetes. However, no association of visceral fat was found in the same analysis [27]. It is also worth mentioning that a national health survey [29] documented that the occurrence of diabetes was more prevalent at a younger age especially obese individuals as compared to their nonobese counterparts. Hence, obesity is the major contribution towards diabetes irrespective of other factors being present or not.

Other statistically significant association was found between age, that is, adult males 45–67 years of age and prediabetes as well as diabetes in our study. Similarly, this trend was also observed in studies done in the USA [6, 34] and China [35, 36]. Another study conducted in Jeddah [17] concluded that age was found to be the strongest predictor for diabetes and prediabetes in males followed by obesity. A Turkish study [31] revealed that the prediabetic stage can start even from the age 20 years and above. In the same context, there were cases of prediabetes in the age group of 20–44 in our study. Hence, this remarkable finding gives us a message to initiate preventive measures and health education or diabetes as the early age so as to prevent our population from the vast health effects and later complications of this disease. Standardized age-sex prevalence was estimated for diabetes and prediabetes based on official national population survey 2017.

Our study population had an average HbA1c level of 5.69, which is within normal ranges; although many of them had an HbA1c levels less than 6.5%, while 10 males had HbA1c levels more than 10%. This could have been due to the presence of more normal-prediabetic or with good glycemic control participants as compared to the diabetic ones. This criterion for diabetes had been suggested previously as an authentic diagnostic entity [37, 38]. HbA1c level between 5.7 and 6.4% was also found in Japanese [39]; however, many previous studies also used fasting blood glucose levels as their recommended diagnostic approaches [38, 40]. The management of poor glycemic control requires lifestyle interventions as its first line of action [37, 38] which includes improving dietary habits and increasing physical activity thereby controlling obesity [41]. These benefits of lifestyle modification were confirmed from Saudi [37], Asian [42, 43], Chinese [44], Americans [41], Finnish [45], and Swedish [46] population. The second resort is the use of antidiabetic agents in combination with physical activity and dietary control; these include biguanides (metformin), thiazolidinediones, *α*-glucosidase inhibitors, the GLP-1 analogues, and insulin secretagouges [38].

Our study also concluded that majority of the diabetics were married. Individuals from Florida demonstrated a significant relationship between marital status and diabetes, married being more at risk for prediabetes as well as diabetes [6]. While in a study [23], men living alone were more prone to diabetes. Even though no significant association was predicted among marital status and diabetes in Bangladesh [47] and Iran [48]. The companionship after getting married can affect both ways, may encourage an individual to stay away from diabetes or may contribute in it. Due to increased responsibility after marriage, people usually do not take time out for their physical fitness and personal grooming which can ultimately lead to obesity and greater risk of diabetes and prediabetes. In the same context, in Greek adults, marital status was found significantly associated with obesity [49] in contrast of Malaysians [50] and Americans [51] who observed higher levels of physical activity after marriage. Hence, such nonmodifiable risk factors can also be controlled to some extent.

We found that education attainment was also significantly associated with diabetes with 58% of the study population with university level education. When Chinese men having high educational level were compared with those having low educational, the latter group was found to have a higher risk of diabetes among men. [32]. Similar findings were reported from Sweden [52], Europe [53], Ethiopia [54], and Bangladesh [47] and our findings. A study done for the assessment of knowledge regarding diabetes showed that men were two times more likely to have better acquaintance regarding as compared with women [55], hence demonstrating some effect of education level on the disease under consideration.

No clear indication for job status as well as smoking association was evident from our data as only 7 individuals in our study were smokers while 1 was unemployed. Likewise, the findings from the studies conducted in China [56] and Turkey [57] were consistent with our findings. However, smoking was found to be significantly associated with diabetes in previous studies [23, 58]. Another study conducted in Jeddah described that single point estimations might miss relationships between variables, as they are unable to cover past practices. This explanation can also be extended to smoking as well as job status [17]. Related or not, smoking cessation efforts can help individuals in reducing their risks of developing other chronic diseases. Regarding job status, Florida reported high occurrence of diabetes in those with low income [6], while less diabetes was seen in military workers due to their jobs demanding high physical activities [59]. Our study compared civilian jobs with unemployment as soldiers were minimal; therefore, job type and its relation with diabetes could not be properly established. The above risk factors whether significantly related or not showed some association with diabetes at some point. Therefore, more detailed and constant evaluation of these is required to establish any possible connection.

## 5. Limitations and Strengths

Our study targeted the actual candidates for diabetes in accordance with the age group and presence of other risk factors. The data was collected by random sampling to make our study generalizable to the population of the country since we did not exclude any participant based on ethnicity or religion. However, this study was particularly done for male gender and so females were not included, and therefore, cross gender differences could not be compared. The age group used was in consistence with the previous published literature; however, both extremes could have been enhanced in order to see the risk factors in young age as well as in elderly. The main portion of prediabetes and diabetes type 2 is however found in the age group used in our study, hence making this a representative sample.

The evaluation of obesity using BMI and waist circumference was done according to the set standards of WHO. Additionally, trained physicians and nurses collected data and anthropometric measurements in order to make the findings genuine together with setting standards for future research. For labeling an individual with diabetes, HbA1c was used which is an accurate measure and also has been used and recommended by WHO as well as by the previous literature. It would have been better if cutoff values for these measurements would be according to Saudi population as various populations may require different cutoffs depending on the individuals residing there. This might contribute towards limiting our data in terms of generalizability. Moreover, other risk factors as highlighted by previous studies, including associated illnesses, family history, dietary habits, and level of physical activity, should also be taken into account while considering risk factors for DM and prediabetes.

## 6. Conclusion

Diabetes mellitus along with its chronic complications has now become serious public health concern. Individuals having prediabetes are becoming diabetic at a faster conversion rate. Therefore, it has become extremely important to hinder this process at an early stage. Our study has identified the important risk factors that are prevailing within the population especially among male Saudi population. The statistically significant predictors for prediabetes and diabetes as evaluated by our study include age, that is, more than 45 years, presence of obesity as measured by BMI and waist circumference, marital status, and educational attainment with exception to job status and smoking habits as they were not significant.

These findings are consistent with previous literature with a free region-related contradictions. Hence, taking into consideration the burden it carries as well as the coping mechanism, which has been in process worldwide, Saudi population also needs such guidelines and strategies to refrain them from the hazards of this disease. Such estimations will aid health practitioners and policy makers to dedicate full efforts in establishing preventive strategies and promoting primary health care for individuals and families in order to hamper the ongoing transmission of diabetes within the families. Nonmodifiable risk factors are difficult to eradicate; however, modifiable factors can be altered. The current scenario of prediabetes and diabetes in Saudi population has been estimated by many studies, before it reaches an epidemic threshold, the task force for the prevention, early diagnosis, prompt treatment, protection against its complications, and health education should be activated.

## 7. Implications and Recommendations

Although Saudi population has been affected by prediabetes and diabetes since long, current revelation statistics should be considered essential in order to initiate preventive and management plans. Primary health care units can play a vital role as almost all the people visit these centers in a way or other. Health education, counseling regarding its risk factors, modifying them along with, constant support, surveillance, encouragement, and proper monitoring should be performed. We know that prediabetic stage usually involves younger age groups, so educational institutes and workplaces should also be utilized as a mode to spread the information. Since, men were found to have more knowledge regarding diabetes, as they are the head of the family, being dominant in the society should be made responsible for protecting their family and people around from adverse health effects. Therefore, social media can be used for this purpose. Secondly, local cutoffs for the local population according to the background, dietary habits, and body characteristics should be established to generalize the findings. Models can be developed from the initial process of taking history to noninvasive risk factor estimation to blood tests and ultimately diagnosing, managing, and preventing its complications.

Data regarding other risk factors like family history, physical activity, and dietary habits should be collected and analyzed. Clustering of various illnesses would help in predicting the common patterns of diseases in which they occur. During the regular health checkups, glycemic control along with the screening for possible complications including neuropathy, nephropathy, retinopathy, and other associated illness and conditions should also be evaluated. In future, risk factors should be compared based on gender, ethnicity, regions, and so on to understand the pathophysiology and any other incomplete connection that may ultimately help in curing this disease and hence managing the disease in a better way.

## Figures and Tables

**Table 1:** General characteristics of participants by diabetic classification in male Al-Kharj study population.

	Total (*N* = 381)	Total nondiabetes (*N* = 346)	Total nondiabetes		Diabetes (*N* = 35)	*P* value for trend^∗∗^
			Nondiabetes (*N* = 241)	Prediabetes (*N* = 105)		
^∗^ *HbA1C*	5.69 (1.12)	5.44 (0.36)	5.25 (0.26)	5.86 (0.17)	8.17 (2.36)	<0.0001
^∗^ *Age in years*	31.42 (9.37)	30.16 (8.17)	28.74 (7.71)	33.43 (8.31)	43.83 (11.35)	<0.0001
*Age groups* (*years*)						<0.0001
18–24	25.72 (98)	27.46 (95)	31.95 (77)	17.14 (18)	8.57 (3)	
25–44	62.99 (240)	65.32 (226)	63.07 (152)	70.48 (74)	40 (14)	
45–60	11.29 (43)	7.23 (25)	4.98 (12)	12.38 (13)	51.43 (18)	
^∗^ *Body mass index in kg*/*m^2^* (*BMI*)	28.75 (6.94)	28.58 (7.03)	27.51 (6.90)	31.02 (6.74)	30.45 (5.71)	<0.0001
*BMI categories*						<0.0001
Normal	31.50 (120)	32.95 (114)	39 (94)	19.05 (20)	17.14 (6)	
Overweight	32.28 (123)	32.66 (113)	33.20 (80)	31.43 (33)	28.57 (10)	
Obese	36.22 (138)	34.39 (119)	27.80 (67)	49.52 (52)	54.29 (19)	
^∗^ *Waist circumference in cm* (*WC*)	99.63 (16.48)	99.08 (16.71)	96.27 (15.78)	105.75 (17.03)	105.24 (12.87)	<0.0001
*Waist circumference*						<0.0001
≤102 cm	64.04 (244)	66.47 (230)	72.61 (175)	52.38 (55)	40 (14)	
>102 cm	35.96 (137)	33.53 (116)	27.39 (66)	47.62 (50)	60 (21)	
*Smoking status*						0.289
Never smoker	66.93 (255)	66.47 (230)	68.88 (166)	60.95 (64)	71.43 (25)	
Ex-smoker	7.87 (30)	7.51 (26)	7.88 (19)	6.67 (7)	11.43 (4)	
Current smoker	25.20 (96)	26.01 (90)	23.24 (56)	32.38 (34)	17.14 (6)	
*Marital status*						<0.0001
Married	57.48 (219)	55.49 (192)	47.72 (115)	73.33 (77)	77.14 (27)	
Single	42.52 (162)	44.51 (154)	52.28 (126)	26.67 (28)	22.86 (8)	
*Education level*						<0.0001
Primary	2.89 (11)	1.45 (5)	0.41 (1)	3.81 (4)	17.14 (6)	
Secondary	25.98 (99)	25.43 (88)	21.99 (53)	33.33 (35)	31.43 (11)	
Intermediate	5.77 (22)	4.91 (17)	4.56 (11)	5.71 (6)	14.29 (5)	
University	58.53 (223)	60.98 (211)	66.80 (161)	47.62 (50)	34.29 (12)	
Postgraduate	6.82 (26)	7.23 (25)	6.22 (15)	9.52 (10)	2.86 (1)	
*Job type*						0.124
Unemployed	8.40 (32)	8.67 (30)	9.96 (24)	5.71 (6)	5.71 (2)	
Civilian	91.08 (347)	90.75 (314)	90.04 (217)	92.38 (97)	94.29 (33)	
Soldier	0.52 (2)	0.58 (2)		1.90 (2)	0	

∗ indicates continuous variables represented as mean (SD). Categorical variable values in each cell are represented as percentage (sample size); ^∗∗^Significance of differences at *p* value < 0.05 evaluated using χ^2^ test for categorical variable and *t*-test or ANOVA for continuous variable. For diabetic versus nondiabetic, all were significant except BMI (*p* value = 0.1278), WC (*p* value = 0.403), smoker (*p* value = 0.422), and job type (*p* value = 0.750), which were nonsignificant. All were significant except smoking status (*p* value = 0.289) at *p* value of 0.05 for diabetic classification (nondiabetic, prediabetic, and diabetic).

**Table 2:** Crude prevalence and trait-specific prevalence of diabetes and prediabetes in male Al-Kharj population.

	Prediabetes (*N* = 105)	95% CI	Diabetes (*N* = 35)	95% CI
*Overall*	**27.56**	23.05–32.07	**9.19**	6.27–12.10
*Age* (*years*)				
18–24	18.37	10.64–26.10	3.06	0.38–6.50
25–44	30.83	24.96–36.71	5.83	2.85–8.81
45–60	30.23	16.30–44.17	41.86	26.89–56.83
*BMI*				
Normal	16.67	9.95–23.38	5	1.07–8.93
Overweight	26.83	18.94–34.72	8.13	3.27–12.99
Obese	37.68	29.54–45.82	13.77	7.98–19.56
*Waist circumference*				
≤102 cm	22.54	17.27–27.81	5.74	2.80–8.67
>102 cm	36.50	28.38–44.61	15.33	9.25–21.40
*Smoking status*				
Never smoker	25.10	19.75–30.45	9.80	6.14–13.47
Ex-smoker	23.33	7.8938.78	13.33	0.92–25.75
Current smoker	35.42	25.77–45.06	6.25	1.37–11.13
*Marital status*				
Married	35.16	28.80–41.52	12.33	7.95–16.71
Single	17.28	11.42–23.14	4.94	1.58–8.30
*Education level*				
Primary	36.36	6.45–66.27	54.55	23.59–85.51
Secondary	35.35	25.85–44.85	11.11	4.87–17.36
Intermediate	27.27	8.16–46.38	22.73	4.75–40.71
University or postgraduate	24.10	18.75–29.44	5.22	2.44–8.00
*Job type*				
Unemployed	18.75	4.97–32.53	6.25	2.30–14.80
Civilian	27.95	23.21–32.70	9.51	6.41–12.61
Soldier	100^∗^	*—* ^∗^	0	*—*

^∗^Estimate based on all values in prediabetes class and soldier.

**Table 3:** Prevalence (%) of prediabetes and diabetes adjusted to national population of Saudi Arabia.

	For prediabetes			For diabetes		
Age groups (years)	Male (*N* = 381)	Female (*N* = 638)	Both (*N* = 1019)	Male (*N* = 381)	Female (*N* = 638)	Both (*N* = 1019)
18–24	5.17	4.01	16.52	0.86	0.38	2.25
25–44	14.52	16.53	32.63	2.75	4.96	8.06
45–60	7.49	11.31	37.98	10.36	9.43	40.20
Overall (18–60 years)	27.17	31.84	**29.46**	13.97	14.76	**14.36**
Overall (25–60 years)	18.25	19.85	19.04	30.62	38.43	34.46

**Table 4:** Regression modeling for diabetic classification and its risk factors in male Al-Kharj population.

			By diabetic classification			
	Unadjusted OR diabetes (versus total nondiabetes)	Adjusted OR^∗^ diabetes (versus total nondiabetes)	Unadjusted OR		Adjusted OR^∗^	
			Prediabetes (versus nondiabetes)	Diabetes (versus nondiabetes)	Prediabetes (versus nondiabetes)	Diabetes (versus nondiabetes)
*Age* (*years*)						
18–24	1	—	1	1	—	—
25–44	**1.91** (0.56–6.48)	—	**2.08** (1.16–3.73)	2.36 (0.66–8.47)	—	—
45–60	**13.67** (4.25–43.99)	—	**4.63** (1.81–11.83)	**38.5** (9.83–150.79)	—	—
*BMI*						
Normal	1	1	1	1	1	1
Overweight	1.63 (0.61–4.33)	0.99 (0.39–2.47)	**1.94** (1.03–3.64)	**1.96** (0.68–5.62)	1.74 (0.91–3.31)	1.24 (0.38–3.99)
Obese	**2.75** (1.14–6.67)	1.52 (0.64–3.59)	**3.65** (2.00–6.67)	**4.44** (1.68–11.72)	**3.29** (1.77–6.11)	2.86 (0.98–8.37)
*Waist circumference*						
≤102 cm	1	1	1	1	1	1
>102 cm	**2.67** (1.40–5.08)	1.26 (0.59–2.68)	**2.41** (1.50–3.88)	**3.98** (1.91–8.28)	**2.11** (1.29–3.46)	**2.03** (1.07–3.77)
*Smoking status*						
Never smoker	1	1	1	1	1	1
Current smoker or ex-smoker	0.81 (0.40–1.63)	1.14 (0.58–2.25)	1.42 (0.88–2.28)	0.88 (0.40–1.93)	1.42 (0.87–2.32)	1.21 (0.50–2.92)
*Marital status*						
Single	1	1	1	1	1	1
Married	**2.49** (1.16–5.35)	1.01 (0.52–1.95)	**3.01** (1.83–4.97)	**3.70** (1.61–8.47)	**1.85** (1.01–3.40)	0.66 (0.22–1.97)
*Education level*						
Primary	1	1	1	1	1	1
Secondary	**0.20** (0.09–0.44)	0.76 (0.32–1.79)	0.17 (0.02–1.54)	**0.03** (0.01–0.32)	0.37 (0.04–3.68)	0.18 (0.02–2.01)
Intermediate	0.42 (0.16–1.07)	0.96 (0.39–2.35)	0.14 (0.01–1.51)	**0.08** (0.01–0.81)	0.27 (0.02-3.20)	0.27 (0.02-3.81)
University or postgraduate	**0.10** (0.05–0.20)	0.43 (0.15–1.29)	**0.09** (0.01–0.78)	**0.01** (0.01–0.11)	0.16 (0.02–1.51)	**0.06** (0.01–0.63)
*Job type*						
Unemployed	1	1	1	1	1	1
Civilian	1.52 (0.38–6.05)	0.27 (0.06–1.17)	1.79 (0.71–4.51)	1.82 (0.41–8.08)	0.82 (0.31–2.22)	**0.11** (0.02–0.65)

^∗^Adjusted by continuous age variable; bold numbers are indicating significant at *p* value of less than 0.05.
